# Selective Elimination of Senescent Fibroblasts by Targeting the Cell Surface Protein ACKR3

**DOI:** 10.3390/ijms23126531

**Published:** 2022-06-10

**Authors:** Kento Takaya, Toru Asou, Kazuo Kishi

**Affiliations:** Department of Plastic and Reconstructive Surgery, Keio University School of Medicine, Tokyo 160-8582, Japan; mori@ideajapan.com (T.A.); kkishi@a7.keio.jp (K.K.)

**Keywords:** senolysis, fibroblast, atypical chemokine receptor 3, antibody-dependent cellular cytotoxicity, natural killer cell

## Abstract

The accumulation of senescent cells in aging tissues is associated with age-related diseases and functional decline. Thus, senolysis, a therapy aimed at rejuvenation by removing senescent cells from the body, is being developed. However, this therapy requires the identification of membrane surface antigens that are specifically expressed on senescent cells for their selective elimination. We showed that atypical chemokine receptor 3 (ACKR3), a receptor of the CXC motif chemokine 12 (CXCL12) implicated in cancer, inflammation, and cardiovascular disorders, is selectively expressed on the surface of senescent human fibroblasts but not on proliferating cells. Importantly, the differential presence of ACKR3 enabled the isolation of senescent cells by flow cytometry using anti-ACKR3 antibodies. Furthermore, antibody-dependent cellular cytotoxicity assays revealed that cell surface ACKR3 preferentially sensitizes senescent but not dividing fibroblasts to cell injury by natural killer cells. Conclusively, the selective expression of ACKR3 on the surface of senescent cells allows the preferential elimination of senescent cells. These results might contribute to the future development of novel senolysis approaches.

## 1. Introduction

Cellular senescence is a state of irreversible growth arrest caused by stress signals, such as the extreme shortening of telomeres, oxidative damage, activation of oncogenes, and hypoxia [1]. Senescent cells exhibit a larger morphology, different patterns of metabolism and gene expression, and increased neutral β-galactosidase activity compared with proliferating cells [2]. Recent studies have shown that senescent cells exhibit the senescence-associated secretory phenotype (SASP) by secreting a series of inflammatory cytokines, chemokines, growth factors, and matrix remodeling factors, which alter the local tissue environment and contribute to chronic inflammation and age-related diseases, such as cancer [3]. Abnormal accumulation of senescent cells generates an inflammatory environment that contributes to pathologies, such as liver and lung fibrosis, atherosclerosis, diabetes, and osteoarthritis [4,5,6].

For this reason, attention has been focused on the development of strategies to specifically eliminate senescent cells. Studies in genetic mouse models in which senescent cells were selectively removed have shown that the accumulation of senescent cells in senescent tissues promotes senescence-associated weakness and disease [7]. Indeed, the removal of senescent cells from aging mouse tissues promoted longevity and healthy life expectancy and was associated with reduced tumor formation and functional expansion of the renal, cardiovascular, muscular, and adipose systems [8,9].

Thus, with growing evidence that senescent cells have a deleterious effect on age-related diseases, the design of therapies that selectively recognize and eradicate senescent cells has become a major goal in this field. Because senescent cells differ significantly from proliferating cells in their expression patterns, including those of markers and proteins on the cell surface that serve as therapeutic targets, therapeutic interventions targeting these markers are expected to achieve senescent cell-specific elimination.

The purpose of our study was to investigate the expression of atypical chemokine receptor 3 (ACKR3), a candidate cell surface protein that is specifically present in senescent cells. Interestingly, CXCL12 is known to be central to the development of many organs and more critically involved in pathophysiological processes underlying cancer, inflammation, and cardiovascular disorders [10]. Its receptor, ACKR3/CXCR7, is an emerging therapeutic target for these diseases. Cancer-associated fibroblasts (CAFs) from solid human tumors are classified into two subgroups, myofibroblasts (myCAFs) and inflammatory fibroblasts (iCAFs), of which iCAFs exhibit a phenotype similar to that of aging SASP fibroblasts [11]. Recent studies have demonstrated the ability of CXCL12 to influence the prognosis of iCAFs [12]. Therefore, we focused on CXCL12 and its membrane surface receptor, ACKR3, in aging skin fibroblasts and hypothesized that they contribute to SASP regulation and senescent cell-specific removal.

Senescent cells are classically defined as cells that irreversibly arrest cell division after repeated passaging [1], but senescence in cells as a result of stress from ionizing radiation [13] or the anticancer drug doxorubicin [14] has also been analyzed.

Using these senescent cell models, we sought to determine whether ACKR3 is a senescent fibroblast-specific marker and whether it can be preferentially eliminated via antibody-dependent cellular cytotoxicity (ADCC) by natural killer (NK) cells that target it.

## 2. Results

### 2.1. ACKR3 Is Specifically Expressed in Senescent Human Dermal Fibroblasts

**Figure 1:**
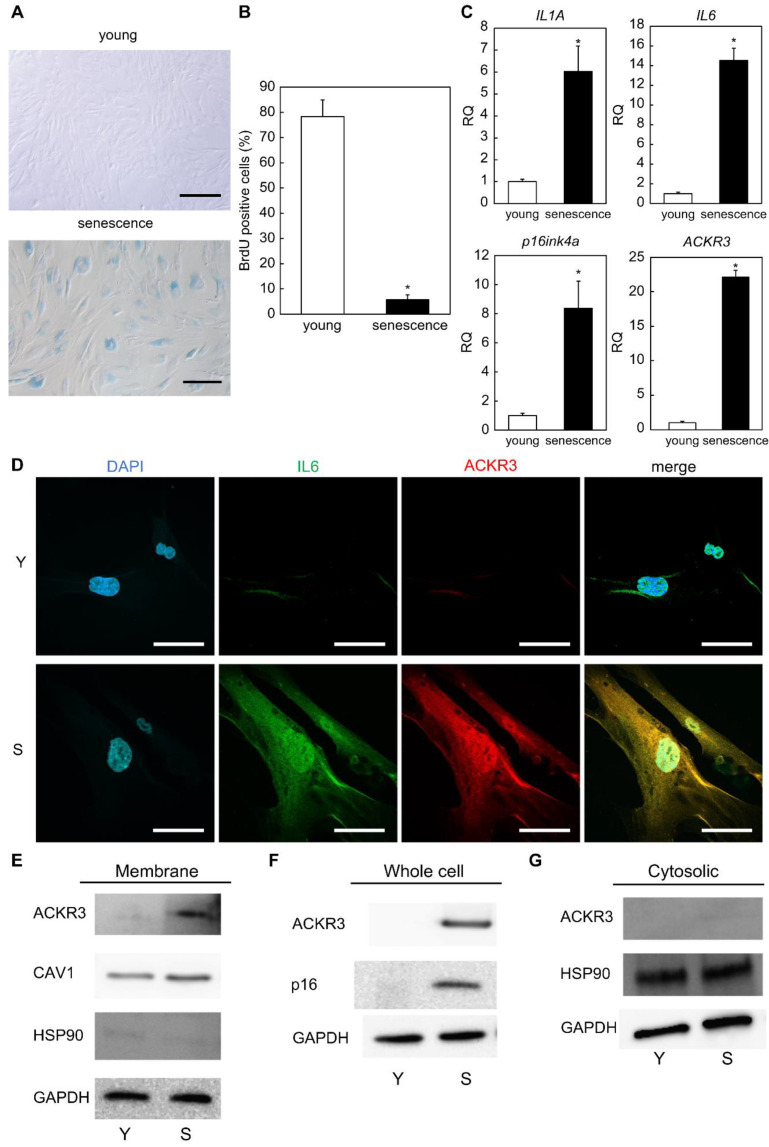
Identification of ACKR3 as a novel senescent cell surface marker protein. (**A**) SA-β-gal staining of proliferating and senescent cells. Bar = 50 µm. (**B**) BrdU absorption by proliferating and senescent cells. (**C**) Real-time quantitative polymerase chain reaction (RT-qPCR) analysis of the expression of senescence-related genes using cell extracts. *GAPDH* was used as the housekeeping gene. (**D**) Immunostaining of IL6 and ACKR3 in young (proliferating) and senescent cells. Bar = 20 µm. Y: young cells. S: senescent cells. (**E**) Western blot analysis of cell extract proteins. (**F**) Western blot analysis of whole-cell lysate proteins. (**G**) Western blot analysis of cytosolic proteins. GAPDH was used as the housekeeping protein. *; *p* < 0.05. RQ; relative quantification. All experiments were repeated in triplicate.

In contrast to the well-known proliferating human dermal fibroblasts (10 population doubling level; PDL), we observed that cells that underwent long-term culture (80–90 PDL) were characterized by a characteristic flattened and expanded morphology and increased SA-β-gal activity (Figure 1A). Furthermore, the analysis of BrdU uptake showed that proliferative activity was significantly lower in senescent cells than in proliferating cells (*p* = 0.0088; Figure 1B). In addition, our real-time quantitative polymerase chain reaction (RT-qPCR) results showed that the expression of *ACKR3* was significantly elevated in senescent cells (*p* = 0.0051). 

At the same time, we found that the levels of gene expression of SASP factors, such as interleukin 1A (*IL1A)* (*p* = 0.049), interleukin 6 (*IL6)* (*p* = 0.0087), and p16ink4a *(CDKN2A)* (*p* = 0.0058) gene levels, which are classically upregulated in senescent cells, were higher in senescent cells compared with proliferating (young) cells (Figure 1C).

We fixed, but not permeabilized, proliferating and senescent skin fibroblasts to detect only proteins present on the outside of the cell membrane in immunostaining. As a result, we noticed that the ACKR3 signal was virtually undetectable in proliferating cells, whereas it was abundant in aging cells in which it was found throughout the cell surface. We further detected that these cells also expressed the SASP factor IL6 (Figure 1D). To investigate the senescent cell surface-specific expression of ACKR3, we isolated membrane-associated and cytoplasmic sol fractions from proliferating and aging cells. Western blot analysis (Figure 1E) revealed that the levels of ACKR3 were definitely elevated in the membrane fraction of senescent but not in proliferating fibroblasts. To monitor the cellular fraction, we included the membrane-associated protein caveolin-1 (CAV1) and the cytosolic sol protein HSP90 (Figure 1E). Western blot analysis of whole-cell lysates similarly showed elevated levels of ACKR3 in senescent cells, as well as an increase in p16, a robust senescent cell marker (Figure 1F). No expression of ACKR3 was observed in Western blots of cytoplasmic fractions (Figure 1G). Thus, ACKR3 is expressed on the plasma membrane of replication-aged human dermal fibroblasts.

To test whether ACKR3 is more commonly increased in senescent cells, we induced senescence by exposing growing human dermal fibroblasts to ionizing radiation (IR); after 10 days, SA-β-gal activity was selectively increased in IR-treated cells (Figure S1A). Western blot and RT-qPCR analysis showed that the protein and gene expression of ACKR3 were upregulated in IR-induced senescent human dermal fibroblast (Figure S1B,C). Treatment with doxorubicin (Dox), another trigger of senescence, also resulted in increased protein and gene expression of ACKR3 abundance in human dermal fibroblasts (Figure S1D–F).

To further test whether ACKR3 is more commonly increased in aging fibroblasts, we induced senescence by exposing growing normal human fetal lung fibroblasts (WI-38) to ionizing radiation (IR). SA-β-gal activity was also selectively increased in IR-treated WI-38 cells. Western blot and RT-qPCR analysis showed that the ACKR3 and *ACKR3* genes were upregulated in IR-induced senescence WI-38, albeit milder than C-12300 (Figure S2B,C).

### 2.2. Contribution of ACKR3 to Senescence

**Figure 2:**
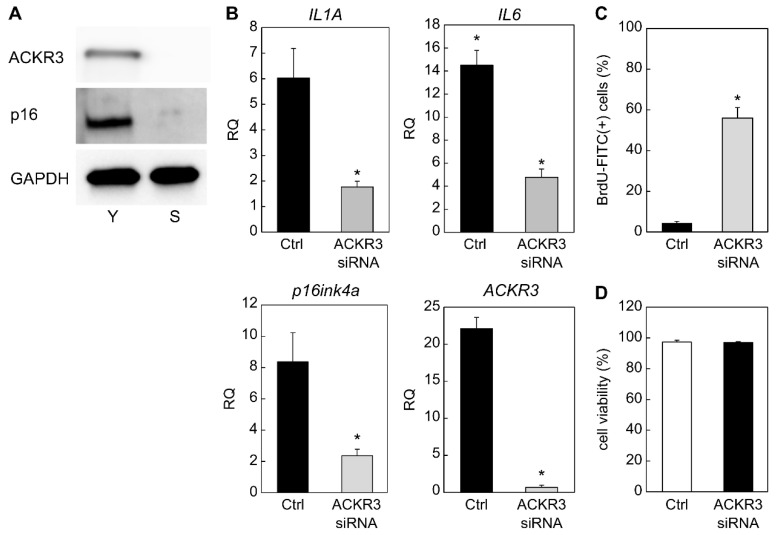
Effect of *ACKR3* siRNA knockdown on senescent cells. (**A**) Western blot analysis to confirm *ACKR3* knockdown and its effect on senescence. (**B**) RT-PCR analysis of the expression of senescence-related genes. *GAPDH* was used as the housekeeping gene. (**C**) Increased BrdU absorption after transfection with *ACKR3* siRNA. (**D**) Cell viability was not altered by ACKR3 siRNA transfection. *; *p* < 0.05. All experiments were performed in triplicate. Ctrl: negative control siRNA.

We further tested whether ACKR3 contributes to the induction of senescence by silencing ACKR3 in senescent fibroblasts and assessing senescence markers. Interestingly, we found that strong silencing of ACKR3 up to 72 h after transfection with siRNA resulted in a marked decrease in the levels of p16, as determined by Western blot analysis (Figure 2A). Similarly, RT-qPCR analysis revealed that strong silencing of *ACKR3* (*p* = 0.0026) was observed in the ACKR3 siRNA group. At the same time, strong silencing of the SASP markers *IL1A* (*p* = 0.034), *IL6* (*p* = 0.026), and senescence marker *CDKN2A* (p16ink4a; *p* = 0.043), a classical biomarker gene for aging, was also observed in the ACKR siRNA group (Figure 2B). Furthermore, we noticed that *ACKR3* silencing promoted BrdU uptake (*p* = 0.01) (Figure 2C), further indicating that ACKR3 contributed to the arrest of cell division, which is characteristic of aging. Cell viability after 72 h of ACKR3 siRNA transfection was unchanged compared to the control (*p* = 0.81) (Figure 2D). In summary, these results indicated that ACKR3 promotes fibroblast senescence.

### 2.3. Specific Selection of Senescent Cells Using Cell Surface ACKR3

**Figure 3:**
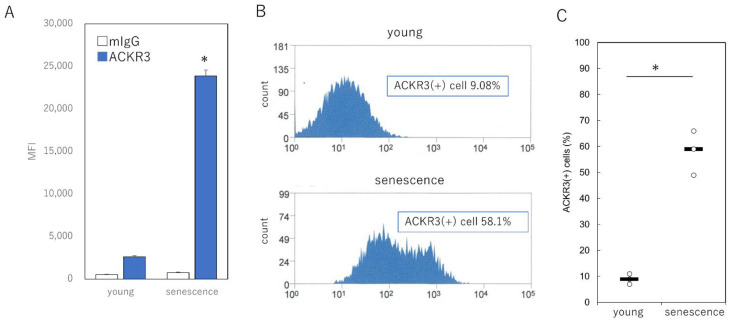
Sorting of senescent cells using an anti-ACKR3 antibody. (**A**) Sorting of ACKR3-positive cells in proliferating and senescent cells. (**B**) Histograms from flow cytometry analysis representing control IgG and anti-ACKR3 antibodies. (**C**) Comparison of the number of ACKR3-positive cells. Each data point represents a young (*n* = 3 total) or senescence (*n* = 3 total) subject; horizontal lines indicate the mean values. * *p* < 0.05. All experiments were performed in triplicate. MFI: mean fluorescence intensity.

Given that ACKR3 has been identified as a cell surface protein of senescent cells, we investigated whether ACKR3 can serve as a suitable selection marker for senescent cells. We labeled proliferating and senescent human fibroblasts without fixation or permeabilization to analyze their cell surface markers using anti-ACKR3 antibodies and control monoclonal IgG. We analyzed labeled samples by flow cytometry to assess the presence of ACKR3 on the cell surface; mean fluorescence intensity analysis revealed that ACKR3 labeling was significantly increased in aged cells compared with young cells during proliferation (*p* = 0.00092) (Figure 3A). Furthermore, we observed that the number of ACKR3-positive cells identified by flow cytometry analysis using ACKR3 increased from only 9.1% in proliferating cells to 58.1% in senescent cells (Figure 3B,C). Collectively, the levels of ACKR3 were markedly elevated on the surface of senescent fibroblasts, and thus ACKR3 can serve as an appropriate marker for the specific isolation of senescent cells.

### 2.4. Selective Elimination of ACKR3-Positive Senescent Cells Using the Antibody-Dependent Cellular Cytotoxicity Assay

**Figure 4:**
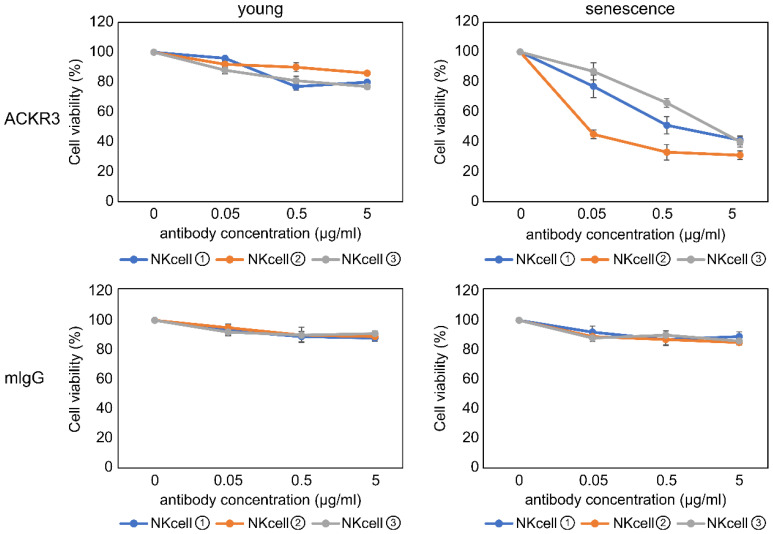
Eliminating ACKR3-positive senescent cells by antibody-dependent cellular cytotoxicity. All experiments were performed in triplicate.

Our initial goal was to devise a method to selectively eliminate senescent cells using surface proteins with different levels of expression. Therefore, after confirming that ACKR3 is present on the plasma membrane surface of senescent cells, we decided to test whether an antibody against ACKR3 could be used to selectively eliminate senescent cells in an ADCC assay. We used concentrations of anti-ACKR3 antibodies (up to 5 μg/mL) to bind the ACKR3 surface marker, thereby labeling proliferating and senescent fibroblasts for ADCC analysis. We then added NK cells from multiple donors to the fibroblast culture to allow NK cells to destroy cells labeled with anti-ACKR3 antibodies. We observed that in all cases, senescent cells showed a strong decrease in viability, which was reduced to as low as approximately 40%, compared with that of proliferating cells (Figure 4). Conversely, ADCC assays using a control rabbit IgG showed no effect on the viability of proliferating or senescent cells (Figure 4).

## 3. Discussion

In summary, ACKR3 was identified as a protein that is strongly upregulated on the plasma membrane of aged human fibroblasts. Furthermore, we found that senescent cells can be selectively targeted and eliminated using anti-ACKR3 antibodies. The high levels of ACKR3 on the exposed surface of senescent cells allow their recognition and elimination by NK cells directed against anti-ACKR3 antibodies. These results highlighted the utility of targeting selective “destruction-marker” proteins that are specifically present on the plasma membrane of senescent cells.

With aging, more senescent cells are observed in mammalian tissues owing to an increase in their numbers or failure of their clearance by apoptosis or immune responses [14,15]. The SASP-promoted induction of inflammation by these immortalized senescent cells clearly contributes to aging [6]. In this regard, the clearance of p16ink4a-positive cells has been reported to lead to significant improvements in lifespan and organ function in vivo [7,16,17]. Hence, removal of senescent cells and suppression of SASP in aging tissues might be an effective approach for the development of therapies that antagonize aging.

Because SASP factors (cytokines, growth factors, and matrix metalloproteinase) disrupt tissue metabolism locally and systemically, they have been associated with disease-enhancing effects of senescent cells that accumulate in aging tissues. For this reason, several drugs have been identified that selectively destroy senescent cells, which is referred to as “senolysis”. The senescent cell eliminators ABT737 [18] and ABT263 [19] are BH3 mimetic inhibitors of antiapoptotic proteins (Bcl-xL, Bcl-2, and Bcl-w) originally developed for cancer therapy; however, the inhibition of Bcl-xL was found to have serious side effects [20]. Other senescent cell eliminators, such as dasatinib and quercetin, cause apoptosis in a subset of senescent cells [21]. One solution focused on the fact that senescent cells differ significantly from proliferating cells in the pattern of expressed proteins, including those on the cell surface that can serve as markers and therapeutic targets. This strategy is similar to that used to selectively eliminate cancer cells [22]. So far, DPP4 [23] and PLAUR [24] have been identified as membrane proteins that are specifically expressed in senescent cells. In this context, the identification of ACKR3 as a targetable senescence marker can complement interventions aimed at eliminating senescent cells.

The detection of novel biomarkers of senescence at the cell surface is becoming an increasingly attractive proposition for a variety of reasons. The definition of a marker is that senescent cells expressing a specific marker on their surface can be easily identified and isolated by flow cytometry without compromising membrane integrity and cell viability [25]. Second, and most importantly, surface molecules have potential uses in vivo, where they can be used as therapeutic targets for drug administration due to the clearance of senescent cells in age-related disorders [26]. Third, senescence markers expressed on cell surfaces can be easily used to classify and analyze specific subpopulations of senescent cells, thus enabling the study of aging heterogeneity [27]. In this study, ACKR3 was strongly upregulated on the surface of senescent cells compared to young cells and was significantly more highly expressed in heterogeneous cell populations. Furthermore, we have achieved cell-specific removal of ACKR3 by ADCC using NK cells in vitro.

ACKR3, also known as RDC1 and CXCR7, was first identified as an orphan G protein-coupled receptor (GPCR) and later described as a high-affinity receptor for CXCL12 and CXCL11 [28,29]. It functions as a scavenger receptor for CXCL12 [30], triggers the β-arrestin pathway [31], and is involved in the regulation of CXCR4 function through the formation of heterodimers [32]. Previous in vitro and in vivo studies have shown that higher levels of ACKR3 are correlated with increased cell proliferation and invasive migration, that is, tumor growth and metastasis [33,34]. However, few reports on its association with senescence have been published to date. The tumor-promoting effects of CAFs, which exhibit a SASP phenotype similar to senescent fibroblasts, have been attributed primarily to CXCL12, which is also an important SASP component [35] that is expressed and secreted by CAFs [36]. Therefore, the suppression of SASP by knockdown of ACKR3 might be common in CAFs.

A limitation of our study was that further research is needed to determine whether ACKR3-targeted NK cells have the necessary safety profile for clinical development. In particular, it is necessary to confirm whether appropriately administered anti-ACKR3 antibodies can infiltrate senescent sites, efficiently target senescent cells, and exert therapeutic effects without noticeable toxicity to the organism. Future iterations of this approach could use combinatorial strategies to maximize efficacy while minimizing side effects [37]. The regulation of SASP by ACKR3 suppression also needs further investigation. For instance, reflecting its importance in embryonic tissues, it has been reported that *ACKR3* knockout mice die perinatally, exhibiting severe defects in cardiovascular, renal, and brain development [38]; thus, such approaches require gene regulation at specific sites and times.

## 4. Materials and Methods

### 4.1. Cell Culture

Normal human dermal fibroblasts (C-12300) were obtained from PromoCell GmbH (Heidelberg, Germany). Normal human fetal lung fibroblast (WI-38) cells were obtained from ATCC (Manassas, VA, USA). Cells were grown in low-glucose Dulbecco’s modified Eagle’s medium (Wako Pure Chemical Industries, Osaka, Japan) supplemented with 10% fetal bovine serum (Thermo Fisher Scientific, Waltham, MA, USA) and 1% penicillin/streptomycin (Thermo Fisher Scientific). Fibroblasts with proliferative senescence or cultured for more than 2 weeks were defined as nonproliferating. For ionizing radiation-induced senescence, cells were exposed to 10 Gy of X-rays by the X-irradiator CellRad (Faxitron, Tucson, AZ, USA) and analyzed 10 days later. For doxorubicin-induced senescence, cells were treated twice with 0.1 μM doxorubicin (Sigma-Aldrich, St. Louis, MO, USA) at 2-day intervals and analyzed after 7 days. Intracellular SA-β-gal activity was assessed using the senescence β-galactosidase staining kit from Cell Signaling Technology (Danvers, MA, USA).

### 4.2. Assessment of BrdU Incorporation in Fibroblasts by Flow Cytometry

Cells were incubated with BrdU at 37 °C for 24 h, collected, and incubated with the BrdU-FITC antibody (BrdUFlowEx FITC Kit; EXBIO Praha, a.s., Vestec, Czech Republic) for 30 min. FlowJo (version 10.2) was used to analyze the data obtained with a flow cytometer (BD Biosciences, Franklin Lakes, NJ, USA).

### 4.3. Immunocytochemistry

Cells were placed on glass slides, fixed in acetone at 25 °C for 5 min, and dried completely before staining. Cells were incubated overnight at 4 °C with either anti-ACKR3 (PA5-27077, Thermo Fisher Scientific) or anti-IL6 (ab6672; Abcam, Cambridge, UK) antibodies diluted 1:100 in phosphate-buffered saline (PBS). After washing 3 times with PBS, slides were incubated with Alexa Fluor 488-conjugated goat anti-rabbit or AlexaFluor555-conjugated donkey anti-goat (Thermo Fisher Scientific) antibodies, respectively, diluted 1:2000 in PBS, for 1 h at 25 °C. After incubation, nuclei were washed 3 times with PBS and counterstained using ProLong gold anti-fade mountant containing 4′,6-diamidino-2-phenylindole (Thermo Fisher Scientific).

### 4.4. RNA Interference and Transfection Method

Lipofectamine 2000 (11668-019; Life Technologies, Invitrogen, Waltham, MA, USA) was used to inject ACKR3 siRNA (109229 and 112094; SilencerTM siRNA, Thermo Fisher Scientific) or negative control siRNA (4390843; Silencer™ Select Negative Control No. 1 siRNA, Thermo Fisher Scientific) into cells. RNA was collected from cells after 72 h, and specific gene knockdown was assessed by RT-qPCR. Cell viability after siRNA transfection was assessed using the MTT Cell Viability Assay Kit (Biotium, Inc., Fremont, CA, USA) as per the protocol recommended by the manufacturer.

### 4.5. RNA Extraction and Reverse Transcription

Total RNA was extracted from cells using a monophasic solution of phenol and guanidine isothiocyanate (ISOGEN; Nippon Gene, Tokyo, Japan) according to the manufacturer’s instructions. Total RNA was mixed with random primers, reverse transcriptase, and a deoxynucleotide mixture (Takara Bio, Tokyo, Japan). For cDNA synthesis, each mixture was incubated in a T100TM thermal cycler (Bio-Rad Laboratories, Inc., Hercules, CA, USA) at 25 °C for 5 min for annealing, 55 °C for 10 min for synthesis, and 80 °C for 10 min for the thermal inactivation of reverse transcriptase.

### 4.6. Real-Time Quantitative Polymerase Chain Reaction

RT-qPCR was performed on an Applied Biosystems 7500 Fast Real-Time PCR System (Thermo Fisher Scientific). A total of 40 cycles were performed, and the fluorescence of each sample was measured at the end of each cycle. Each PCR reaction was performed in 2 major steps: holding the reagents at 95 °C for 3 s (denaturation) and at 60 °C for 30 s (annealing and extension), while in the subsequent melting curve analysis stage, the temperature was increased from 60 °C to 95 °C, and fluorescence was measured continuously. Gene expression was determined using primers for *ACKR3* (assay ID: Hs00664172_s1), *IL6* (Hs00985639_m1), *IL1A* (Hs00174092_m1), and *p16ink4a* (Hs00923894_m1) (all from Thermo Fisher Scientific) and a PCR master mix (Cat. No. 4352042; Applied Biosystems, Foster City, CA, USA) as per the manufacturers’ instructions. *GAPDH* (Hs02786624_g1) was used as a control. The level of gene expression in the proliferating cell population was used as the baseline, and fold change values were determined using the 2-ΔΔCT method as previously reported [39].

### 4.7. Western Blotting

Total protein was extracted from cells and tissues. Tissues were preshredded and treated with collagenase (FUJIFILM Wako Pure Chemical Co., Osaka, Japan). Samples were extracted in lysate buffer: 50 mM Tris-HCl (pH 8.0), 150 mM NaCl, 0.5% Nonidet P40, 0.5% sodium deoxycholate, and phenylmethylsulfonyl fluoride (FUJIFILM Wako Pure Chemical Co.).

To extract membrane and cytosolic proteins, cell lysates were prepared and processed from cells using the Mem-PER™ plus membrane protein extraction kit (Thermo Fisher Scientific) or the Pierce™ cell surface protein isolation kit (Thermo Fisher Scientific).

Each sample (40 μg) was electrophoresed on 10 % polyacrylamide gels (Mini-PROTEAN TGX precast gels; Bio-Rad Laboratories, Inc.) and transferred to polyvinylidene difluoride membranes (Millipore, Bedford, MA, USA) using a Trans-Blot turbo transfer system (Bio-Rad Laboratories, Inc.).

After blocking with 3% nonfat milk for 2 h at 25 °C, membranes were incubated overnight at 4 °C with primary antibodies against ACKR3 (1:200, PA5-27077; Thermo Fisher Scientific), p21 (1:100, ab220206; Abcam), CAV1 (1:1200, sc-53564; Santa Cruz Biotechnology, Santa Cruz, CA, USA), HSP90 (1:100, ab13492; Abcam), and GAPDH (1:2000; Santa Cruz Biotechnology) diluted in blocking solution. The next day, samples were incubated with the following secondary antibodies: donkey anti-goat IgG H&L (horseradish peroxidase; HRP) (ab6885; Abcam), goat anti-rabbit IgG H&L (HRP) (ab205718; Abcam), and goat anti-mouse IgG H&L (HRP) (ab205719; Abcam) at a 1:1000 dilution each for 2 h at 37 °C. After washing, immunoreactive protein bands were visualized using an electrochemiluminescence detection kit (Pierce Biotechnology, Rockford, IL, USA). Images of bands were obtained using a chemiluminescence imager (ImageQuant LAS4000mini; GE Healthcare, Chicago, IL, USA). Image analysis was performed using ImageJ (Ver. 1.53p, National Institutes of Health, Bethesda, MD, USA). Each experiment was repeated three times.

### 4.8. Fluorescence-Activated Cell Sorting (FACS)

Proliferating and senescent human skin fibroblasts were counted using a TC20 cell counter (Bio-Rad Laboratories, Inc.) and washed using FACS buffer (0.5% bovine serum albumin in PBS). After washing, human TruStain FcX (BioLegend, San Diego, CA, USA) was added to block the Fc receptor, and cells were incubated with control IgG (ab172730, Abcam) or an anti-ACKR3 antibody (Thermo Fisher Scientific) for 10 min at 4 °C. Cells were then incubated with an Alexa Fluor 488-labeled rabbit anti-goat antibody (Thermo Fisher Scientific) for 15 min at 4 °C in the dark. Next, 7-amino-actinomycin D (Immunostep, S.L., Salamanca, Spain) was added to the culture and incubated at 4 °C for 15 min to label the photoreceptor cells. FACS analysis was performed using FlowJo (version 10.2). Alexa-Fluor 488-positive cells were counted after the removal of 7-AAD+ cells.

### 4.9. Antibody-Dependent Cellular Cytotoxicity Assay

Proliferating and senescent fibroblasts (5 × 10^4^ cells per well) cultured on 24-well plates were incubated with monoclonal antibodies recognizing different concentrations of control IgG or 0.05, 0.5, and 5 μg/mL anti-ACKR3 antibody for 15 min at 37 °C. NK cells were obtained from three different donors from ATCC (Manassas, VA, USA); NK cells (2.5 × 10^5^ cells/well) were added at a ratio of 5:1 (proliferating fibroblasts: NK cells) or 50:1 (senescent fibroblasts; NK cells) and cells were incubated at 37 °C for 4 h. NK cells were removed by washing, fibroblasts were returned to the incubator, and cell viability was analyzed after 24 h using the CellTiter-Glo^®^ 2.0 cell viability assay (Promega, Madison, WI, USA).

### 4.10. Statistical Analysis

Statistical analyses were performed using GraphPad Prism (version 5.0; San Diego, CA, USA) or SPSS 22.0 (Chicago, IL, USA). Single end-point measures were compared using a nonpaired sample *t*-test. Values of *p* < 0.05 were considered statistically significant.

## 5. Conclusions

In summary, our findings identified that the cell membrane-associated protein ACKR3 is a senescence marker and a promising target for therapeutic intervention under conditions where the elimination of senescent cells is desirable.

## Data Availability

The data presented in this study are available on request from the corresponding author.

## Supplementary Materials

The following supporting information can be downloaded at: www.mdpi.com/article/10.3390/ijms23126531/s1.
