# Supplementary material for: Selective Elimination of Senescent Fibroblasts by Targeting the Cell Surface Protein ACKR3
Source: Int J Mol Sci. 2022 Jun 10;23(12):6531. doi: 10.3390/ijms23126531 (PMC9223754; doi:10.3390/ijms23126531)
Supplement: Supplementary file 1 [file ijms-23-06531-s001.zip › ijms-1758717-supplementary.pdf]

Supplementary legends

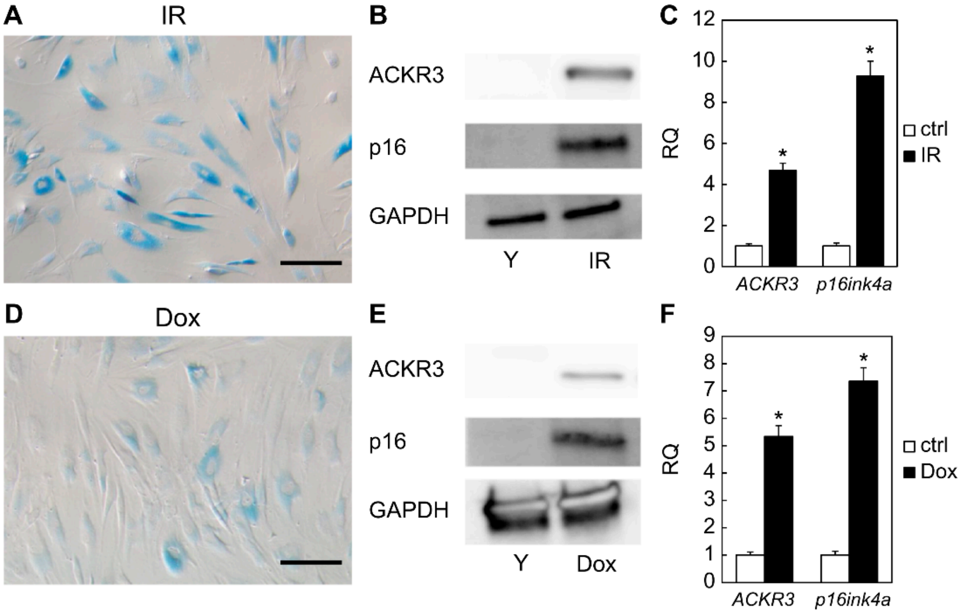

Figure S1 ACKR3 expression in different cellular senescence stress models. (a) SA-β-gal staining of IR-induced senescent cells. Bar = 50 μm. (b) Western blot analysis of whole-cell lysate proteins extracted from IR-induced senescent cell. (c) Real-time quantitative polymerase chain reaction (RT-qPCR) analysis of the expression of senescence-related genes using cell extracts. GAPDH was used as the housekeeping gene. (d) SA-β-gal staining of doxorubicin-induced senescent cells. Bar = 50 μm. (e) Western blot analysis of whole-cell lysate proteins extracted from IR-induced senescent cell. (f) RT-qPCR analysis of the expression of senescence-related genes using cell extracts. GAPDH was used as the housekeeping gene. *; $P < 0.05$. IR; ionizing radiation. Dox; doxorubicin. RQ; relative quantification. All experiments were repeated in triplicate.

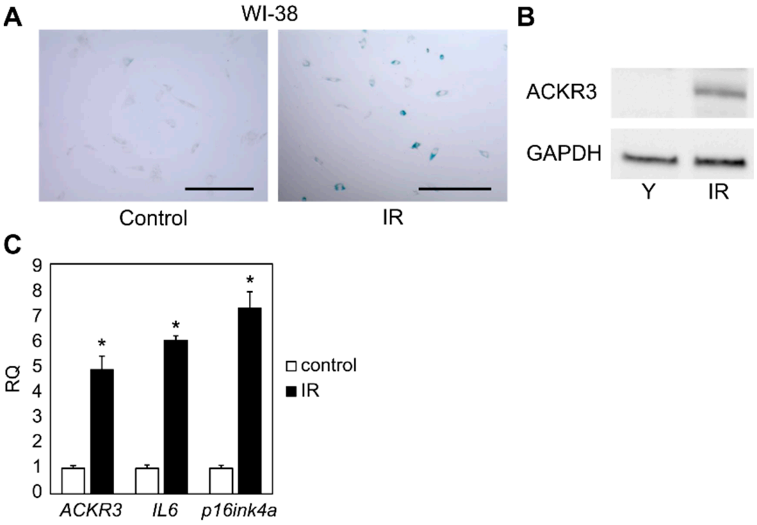

Figure S2 Expression of ACKR3 in different human fibroblasts (WI-38). (a) SA-β-gal staining of IR induced WI-38 senescent cells. Bar = 50 μm. (b) Western blot analysis of whole-cell lysate proteins extracted from IR induced WI-38 senescent cell. (c) Real-time quantitative polymerase chain reaction (RT-qPCR) analysis of the expression of senescence-related genes using cell extracts. GAPDH was used as the housekeeping gene. *; $P < 0.05$. RQ; relative quantification. All experiments were repeated in triplicate.
